# Effects of differential measurement error in self-reported diet in longitudinal lifestyle intervention studies

**DOI:** 10.1186/s12966-021-01184-x

**Published:** 2021-09-16

**Authors:** David Aaby, Juned Siddique

**Affiliations:** Department of Preventive Medicine, Northwestern University, Chicago, Israel

**Keywords:** Bias, Sample size, Coverage, Behavior, Intervention, Clinical trial

## Abstract

**Background:**

Lifestyle intervention studies often use self-reported measures of diet as an outcome variable to measure changes in dietary intake. The presence of measurement error in self-reported diet due to participant failure to accurately report their diet is well known. Less familiar to researchers is *differential* measurement error, where the nature of measurement error differs by treatment group and/or time. Differential measurement error is often present in intervention studies and can result in biased estimates of the treatment effect and reduced power to detect treatment effects. Investigators need to be aware of the impact of differential measurement error when designing intervention studies that use self-reported measures.

**Methods:**

We use simulation to assess the consequences of differential measurement error on the ability to estimate treatment effects in a two-arm randomized trial with two time points. We simulate data under a variety of scenarios, focusing on how different factors affect power to detect a treatment effect, bias of the treatment effect, and coverage of the 95% confidence interval of the treatment effect. Simulations use realistic scenarios based on data from the Trials of Hypertension Prevention Study. Simulated sample sizes ranged from 110-380 per group.

**Results:**

Realistic differential measurement error seen in lifestyle intervention studies can require an increased sample size to achieve 80% power to detect a treatment effect and may result in a biased estimate of the treatment effect.

**Conclusions:**

Investigators designing intervention studies that use self-reported measures should take differential measurement error into account by increasing their sample size, incorporating an internal validation study, and/or identifying statistical methods to correct for differential measurement error.

## Introduction

Lifestyle intervention studies—which aim to change a participant’s weight or eating behavior—often use self-reported measures of diet, such as interviewer-assisted 24-hour dietary recalls or food frequency questionnaires. These measures are prone to error for various reasons including poor quantification of portion sizes and social desirability [1]. More reliable and accurate measures, such as recovery biomarkers, require a 24-hour urine collection, and are expensive and cumbersome for participants to collect. Thus self-reported measures are commonly used as a surrogate for the true quantity of interest.

Measurement error in intervention studies can result in biased estimates of the treatment effect and reduced power to detect treatment effects [2]. Measurement error interferes with analyses to determine if an intervention is effective and limits the ability of researchers to design and implement effective interventions to reduce behavioral risk factors. Thus, the presence of measurement error may lead researchers to adopt or discard interventions that are actually (in)effective.

Most measurement error research has focused primarily on problems associated with measurement error in predictor variables [3], particularly those situations where an exposure is measured with error, thus attenuating or distorting the relationship between exposure and outcome. Less work has been done investigating the implications of measurement error in outcome variables in a longitudinal intervention setting. Longitudinal dietary intervention studies involve repeated dietary assessments over time and produce unique measurement error issues that are not encountered in cross-sectional studies. Participants may modify their reporting behavior to appear compliant with dietary recommendations of the study [4], or they may attempt to reduce interview duration and reporting difficulty during follow-up assessments by omitting items or by erroneously reporting foods that are easier to measure or describe [5]. Alternatively, their accuracy may improve over time due to training in portion size assessment and a more general awareness of their dietary intake [6, 7].

Differential measurement error is where the nature of measurement error (bias and precision) differs over time and/or by treatment condition [8, 9]. In terms of bias, participants may (1) become more accurate in their reports of diet due to improved self-monitoring; (2) misreport their diet in order to appear compliant with the intervention; or (3) report with the same accuracy as seen at baseline. Similarly, the precision of reporting may increase, decrease, or stay the same as at baseline. These changes in bias and precision may differ by treatment condition. While a biased treatment effect is clearly undesirable, reduced power due to additional variability is not a trivial matter in lifestyle interventions, where effect sizes tend to be small and the additional variation due to measurement error can result in a failure to detect a treatment effect.

In this paper, using the setting of a longitudinal clinical trial, we use simulation to derive how various forms of differential measurement error influence sample size, bias, and coverage of the 95% confidence interval when estimating treatment effects. We provide recommendations for investigators when designing intervention trials that use dietary intake as an outcome variable.

## Methods

In this section, we describe a simulation study to assess the consequences of outcome measurement error and differential measurement error on the ability to estimate treatment effects. Our simulation-based scenarios reflect the settings of intervention studies where diet-based outcomes are measured repeatedly over time in both a treatment and control group. The analysis model is a covariance pattern regression model [10] where the outcome is modeled as a function of time, a treatment by time interaction, and where an unstructured covariance matrix is used to estimate the variance-covariance parameters.

We examine 1) Differential measurement error with respect to time which is reflected in differences in measurement error variability between baseline and follow-up as well as differences in over/under reporting at baseline as compared to follow-up; 2) Differential measurement error with respect to treatment condition, with participants in the treatment group having different measurement error (variability and bias) as compared to those in the control group. To explore these settings, we simulate data under a variety of scenarios, focusing on how different factors influence: the power to detect the treatment effect, the bias of the treatment effect, and the coverage of the 95% confidence interval of the treatment effect.

Let *z*_*ij*_ be the true value (i.e. true dietary intake) of the quantity we wish to measure on participant *i*,*i*=1,…,*N* at time *j*,*j*=0,1. This quantity is assumed to be measured without error. Let *y*_*ij*_ be the observed outcome of interest measured with error. That is, *y*_*ij*_ is *z*_*ij*_ measured with error, such as a self-reported dietary measure. Let *d*_*i*_ be an indicator as to whether a participant has been randomized to the treatment group (*d*_*i*_=1) or the control group (*d*_*i*_=0). The variable *t*_*ij*_ indicates the time points at which the quantities are measured, a baseline measurement (*t*_*ij*_=0) and a follow-up measurement (*t*_*ij*_=1). Finally, let *n*_*d*_ denote the sample size in each treatment and control group.

The distribution of *z*_*ij*_ has the following form:

1 $$ z_{{ij}} = \beta_{0} + \beta_{1}t_{{ij}} + \beta_{2}\left(t_{{ij}} \times d_{i}\right) + \varepsilon_{{ij}}  $$

where *β*_0_ is an intercept term and *β*_1_ is the effect of time. We assume no differences in intervention conditions at baseline, and thus do not include a main effect for treatment. The regression coefficient *β*_2_ is the estimand of interest, the expected true difference in change over time between the two treatment conditions. In all of our simulations, we fix the values of the regression coefficients in Eq. (1) at the values listed in Table 1. That is, *β*_0_=8.21,*β*_1_=−0.037, and *β*_2_=−0.25. These values are based on data from the Trials of Hypertension Prevention (TOHP) study (described below).

**Table 1:** Simulation Study Parameter Values

Parameter	TOHP Value	Range of Simulation Values	Equation
*β*_0_	8.21	Fixed	2.1
*β*_1_	-0.037	Fixed	2.1
*β*_2_	-0.25	Fixed	2.1
$\sigma ^{2}_{z}$	0.17	Fixed	2.2
*ρ*	0.5	Fixed	2.2
*γ*_0_	5.29	Fixed	2.3
*γ*_1_	0.09	Fixed	2.3
*γ*_2_	0.33	1, 0.33	2.3
*γ*_3_	-0.006	−1.5 to 1.5 by 0.01	2.3
*γ*_4_	-0.034	−0.10 to 0.05 by 0.001	2.3
*λ*_1_	1.86	Fixed	2.4
*λ*_2_	1.17	0.5 to 2.5 by 0.1	2.4
*λ*_3_	1.32	0.5 to 2.5 by 0.1	2.4

In Eq. (1), *ε*_*ij*_ is a random error term with distribution *ε*_*ij*_∼*N*(0,*Σ*_*z*_). The variance-covariance matrix *Σ*_*z*_ is

2 $$ \Sigma_{z} \; = \; \left[ \begin{array}{cc} \sigma^{2}_{z} & \rho \sigma^{2}_{z} \\ \rho \sigma^{2}_{z} & \sigma^{2}_{z} \end{array} \right]  $$

where $\sigma ^{2}_{z}$ is the variance at baseline and follow-up and *ρ* is the correlation between baseline and follow-up. Again, these values are listed in Table 1 and are fixed across all simulation scenarios ($\sigma _{z}^{2}=0.17, \rho =0.5$).

To generate *y*_*ij*_ we add measurement error to the *z*_*ij*_ values as follows:

3 $$\begin{array}{*{20}l} {}y_{{ij}} &= \gamma_{0} + \gamma_{1}\left(d_{i} \times t_{{ij}}\right) + \gamma_{2}z_{{ij}} + \gamma_{3}\left(z_{{ij}}\times t_{{ij}}\right) \\&\quad+ \gamma_{4}\left(z_{{ij}} \times t_{{ij}} \times d_{i}\right) + \delta_{{ij}} \end{array} $$

where *γ*_0_ is an intercept term reflecting the overall difference between self-report and true intake at baseline for a given value of *z*_*ij*_,*γ*_1_ is the additional change in intercept between treatment conditions at follow-up, *γ*_2_ is the slope of the regression of *y*_*ij*_ on *z*_*ij*_ at baseline that reflects how *y*_*ij*_ varys as a function of true intake at baseline, *γ*_2_+*γ*_3_ is the slope in the control group at time 1, and *γ*_4_ is the difference in slopes between the treatment and control groups at time 1.

The error term *δ*_*ij*_ in Eq. (3) is normally distributed with *δ*_*ij*_∼*N*(0,*Σ*_*y*_). The variance-covariance matrix *Σ*_*y*_ is

4 $$ \Sigma_{y} \; = \; \left[ \begin{array}{cc} \lambda_{1}\sigma^{2}_{z} & \rho \sigma^{2}_{z} \\ \rho \sigma^{2}_{z} & \lambda_{1}\lambda_{2}\lambda_{3}^{d_{i}}\sigma^{2}_{z} \end{array} \right]  $$

where *λ*_1_ is a factor for inflating variance at every time point, *λ*_2_ is a factor for inflating variance at follow-up, and *λ*_3_ is a factor for inflating variance in the treatment group at follow-up. Eq. (4) allows for repeated measures on participant *i* to be correlated and can also allow for heterogeneous variances.

Equations (1) through (4) provide a very flexible framework for simulating data and incorporate a number of scenarios for simulating differential measurement error. Table 2 summarizes—in terms of our model—various types of measurement error and how the parameters are set or varied in our simulation scenarios. For example, when the parameters *γ*_0_,*γ*_1_,*γ*_3_,*γ*_4_=0 and *γ*_2_=1 in Eq. (3), *y*_*ij*_ follows a classic measurement error model, where *y*_*ij*_ is an unbiased measure of *z*_*ij*_, but measured with additional variability (Table 2, row 1). We focus on the last three rows of Table 2: differential measurement with respect to time, differential measurement error with respect to treatment, and differential measurement error with respect to time and treatment.

**Table 2:** Different measurement error scenarios described in terms of Eqs. (3) and (4) where *z*_*ij*_ is true intake for participant *i* at time *j*. *y*_*ij*_ is *z*_*ij*_ measured with error

Type of	Model in terms of	Varying sim	Varying sim
Measurement	Equations (2.3) and (2.4)	parameters	parameters
Error		(bias)	(variance)
Classical ME	*y*_*ij*_=*z*_*ij*_+*δ*_*ij*_	NA	NA
Systematic ME (non-DME)	*y*_*ij*_=*γ*_0_+*γ*_2_*z*_*ij*_+*δ*_*ij*_	NA	NA
DME w.r.t. time	*y*_*ij*_=*γ*_0_+*γ*_2_*z*_*ij*_+*γ*_3_(*z*_*ij*_×*t*_*ij*_)+*δ*_*ij*_	*γ*_3_	*λ*_2_
DME w.r.t. tx	*y*_*ij*_=*γ*_0_+*γ*_1_(*d*_*i*_×*t*_*ij*_)+*γ*_2_*z*_*ij*_	*γ*_4_	*λ*_3_
	+ *γ*_4_(*z*_*ij*_×*t*_*ij*_×*d*_*i*_)+*δ*_*ij*_		
DME w.r.t. time and tx	*y*_*ij*_=*γ*_0_+*γ*_1_(*d*_*i*_×*t*_*ij*_)+*γ*_2_*z*_*ij*_	*γ*_3_,*γ*_4_	*λ*_2_,*λ*_3_
	+ *γ*_3_(*z*_*ij*_×*t*_*ij*_)		
	+ *γ*_4_(*z*_*ij*_×*t*_*ij*_×*d*_*i*_)+*δ*_*ij*_		

Abbreviations: *ME* measurement error; *DME* differential measurement error; *w.r.t.* with respect to; *tx* treatment

In these scenarios, the parameters *γ*_3_ and *γ*_4_ allow for changes in bias at follow-up and by treatment condition, respectively. The parameters *λ*_2_ and *λ*_3_ allow for additional variability at follow-up and within the treatment group, respectively.

Using this simulation framework, and varying the sample size as well as the parameters *γ*_3_,*γ*_4_,*λ*_2_, and *λ*_3_ in Eqs. (3) and (4), we simulate data under a variety of scenarios. Each set of simulations is centered at non-differential measurement error (Table 2, row 2). We then expand our simulations around this central assumption to investigate how differential measurement error impacts estimates of the treatment effect.

To ensure that we are simulating realistic scenarios, we calibrate the simulation parameter values in Eqs. (1) through (4) using data on sodium intake from the Trials of Hypertension Prevention Study (TOHP), a randomized controlled trial of 2811 participants who received lifestyle interventions and nutritional supplement interventions for hypertension prevention [11]. TOHP collected 24-hour recalls as well as urinary sodium on 744 participants, both at baseline and at follow-up. This allows us to posit realistic values for the parameters involving true intake in Eqs. (1) and (2) as well for the parameters involving measurement error in Eqs. (3) and (4).

Table 1 summarizes the parameters estimated from the TOHP data as well as the varying values used in the simulations. The true treatment effect, *β*_2_ in Eq. (1), is -0.25 on the log scale so that at follow-up, participants in the treatment condition have sodium intake (1− exp(−0.25))×100=22*%* less than those in the control group.

We define the *naive* treatment effect as the difference in change from baseline between treatment and control groups using the error prone self-reported values *y*. Using Eq. (3), the naive treatment effect is given by:

5 $$\begin{array}{*{20}l} \Psi^{naive} &=\mathrm{E}(y_{1} - y_{0} \mid d_{i} = 1) - \mathrm{E}(y_{1} - y_{0} \mid d_{i} = 0) \notag \\ &= \gamma_{1} + \beta_{2}(\gamma_{2} + \gamma_{3}) + \gamma_{4}(\beta_{0} + \beta_{1} + \beta_{2}). \end{array} $$

See Section A.1 in the appendix for details. An estimate of the treatment effect is unbiased when *Ψ*^*naive*^−*β*_2_=0.

### Variability simulation

We estimated the naive treatment effect in (5) under a variety of simulation scenarios by varying the parameters in Table 1. We examined how simultaneously increasing measurement error variability at follow-up (*λ*_2_) and increasing measurement error variability in the treatment condition at follow-up (*λ*_3_) increases the required sample size to achieve 80% power. We assume that the trial was powered assuming non-differential measurement error based on existing self-reported data. Thus, the parameter for increased variability across all participants regardless of time point or treatment condition (*λ*_1_) was fixed across all scenarios and equal to 1.86. Calculation of power was based on a two-sample z-test, as defined in Power and sample size of the Appendix.

### Treatment effect simulation

Next, we examined how simultaneously varying the change in slope for the control group at follow-up (*γ*_3_) and varying the change in slope for the treatment group at follow-up (*γ*_4_) increases the bias of the treatment effect in terms of the percent increase in the bias of the treatment effect. We fix the parameters *γ*_1_ and *γ*_2_ to the TOHP values displayed in Table 1. Thus only the *γ* parameters that affect measurement error differentially (i.e. *γ*_3_ and *γ*_4_) influence the percent increase in bias of the treatment effect. In our setting, an increase in slope results in greater self-reported values at follow-up as compared to baseline (or the control group) for a fixed value of true intake.

### Coverage simulation

Finally, we varied both the differential measurement error parameters in Table 2 affecting bias (*γ*_3_,*γ*_4_) and the differential measurement error parameters that affect variance (*λ*_2_,*λ*_3_) to generate different combinations of high/low bias and high/low variance. The parameters that do not affect differential measurement error (*γ*_1_,*γ*_2_,*λ*_1_) were fixed at their TOHP values. We calculated the naive treatment effect and its 95% confidence interval (Appendix Coverage). To compare these scenarios to each other and that based on true intake, we display our results using a forest plot.

The coverage probability of a confidence interval is the proportion of the time that the interval contains the true quantity of interest *β*_2_. Coverage can be affected by both bias and variability and as a result, provides a good summary of how different parameters affecting measurement error can impact estimates of the treatment effect. Let *Ψ*_*lower*_ and *Ψ*_*upper*_ be the lower and upper endpoints of a 95% confidence interval of an estimate of the naive treatment effect. Ideally, an estimator exhibits *nominal* coverage, such that the coverage of its 95% confidence interval is also 95%. We calculate the coverage of the naive treatment effect as the probability that the true treatment effect lies within the 95% confidence interval of the naive treatment effect. Details are in Coverage of the appendix.

## Results

**Fig. 1:**
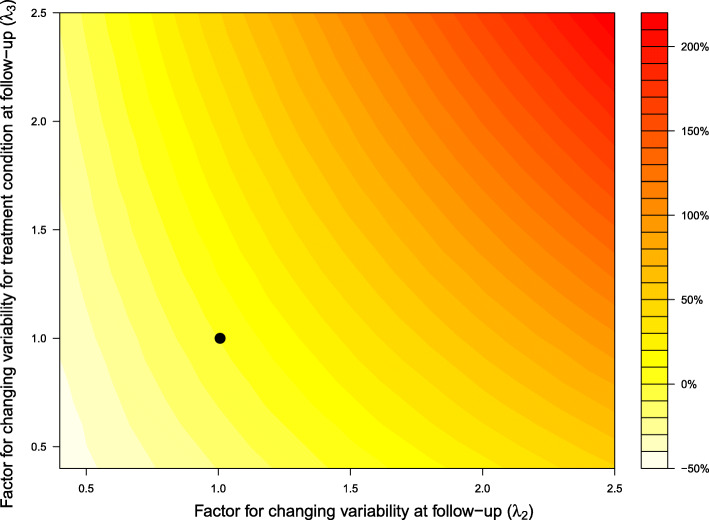
Contour plot for the percent change in sample size required to achieve 80% power to detect a fixed treatment effect across a range of parameters that change measurement error variability differentially with respect to follow-up and treatment condition at follow-up. The x-axis displays values for the parameter for changing variability at follow-up (*λ*_2_), the y-axis displays values for the parameter for changing variability for the treatment condition at follow-up (*λ*_3_). The point plotted at (1,1) corresponds to 0% change in sample size due to no change in variability at follow-up or treatment condition at follow-up

Figure 1 is a contour plot of the percent increase in sample size needed to achieve 80% power to detect a treatment effect. The x-axis displays values for the measurement error parameter for the additional variability at follow-up (*λ*_2_). The y-axis displays values for the measurement error parameter for increasing variability for the treatment condition at follow-up (*λ*_3_). As these parameters increase, so does the sample size needed to achieve 80% power.

Using estimates from the TOHP data, under a scenario of no increased variability at follow-up in both the treatment and control conditions (*λ*_2_=1,*λ*_3_=1), the sample size needed to achieve 80% power is *n*=117 per group (indicated by the black dot in Fig. 1). Differential measurement error with respect to time (*λ*_2_>1,*λ*_3_=1) has a greater impact on required sample size to achieve 80% power than does differential measurement error with respect to treatment (*λ*_2_=1,*λ*_3_>1). For example, under a scenario where there is additional variability at follow-up (*λ*_2_=2) but no additional variability for treatment condition (*λ*_3_=1), the sample size must increase by 65.0% in order to achieve 80% power, which corresponds to a sample size of *n*=193 per group. For scenarios where there is no additional variability at follow-up in the control group (*λ*_2_=1) but additional variability only in the treatment condition at follow-up, (*λ*_3_=2), the sample size must increase by only 32.5%, (*n*=155 per group). For situations where there is both increased variability at follow-up and treatment condition at follow-up, (*λ*_2_=2,*λ*_3_=2), the sample size must increase by 130.8%, which corresponds to a sample size of *n*=270 per group. Under scenarios of decreased variability, the required sample size decreases. For example, when *λ*_2_=.5 and *λ*_3_=.5, the sample decreases by 40% (*n*=70 per group).

**Fig. 2:**
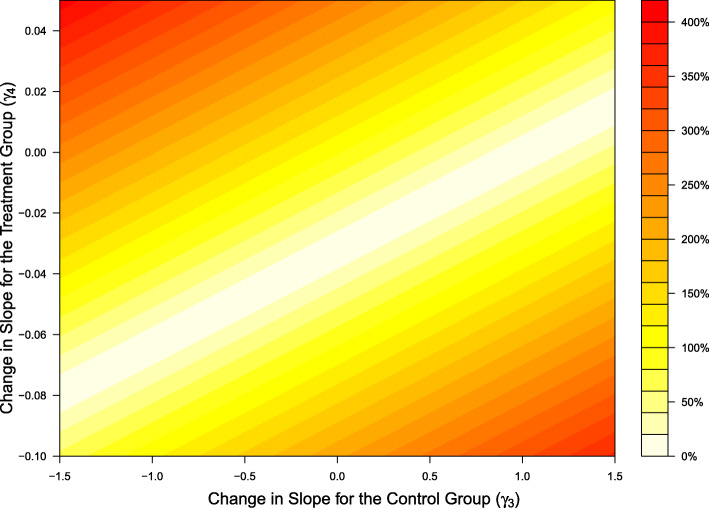
Contour plot for the percent increase in bias across a range of parameters for the slope of the treatment effect. The x-axis displays values for the measurement error parameter for the change in slope for the control group (*γ*_3_), the y-axis displays values for the measurement error parameter for the change in slope for the treatment group (*λ*_4_). Increases in differential measurement error with respect to treatment have a stronger impact on bias than do increases in differential measurement error with respect to time

Figure 2 is a contour plot of the percent increase in bias of the treatment effect for varying values of *γ*_3_—the measurement error parameter for change in slope for the control group (x-axis), and *γ*_4_—the additional change in slope for the treatment group (y-axis). As measurement error increases in the intervention groups, so does the bias of the treatment effect.

Unlike the parameters governing variance, here differential measurement error with respect to treatment (*γ*_4_) does have a substantial effect. For example, when there is no additional change in slope for the treatment group at follow-up (*γ*_4_=0), and a small increase in slope for the control group (*γ*_3_ increases from 1.02 to 1.10), then the bias increases by only 8%, so that the naive treatment effect reflects a 23.7% reduction in sodium intake in the treatment group versus the control group (as compared to the true treatment effect of a 22% reduction). When there is no additional change in slope for the control group at follow-up (*γ*_3_=0) and a small increase in slope for the treatment group at follow-up (*γ*_4_ increases from -0.032 to -0.05), the bias increases by 56.5% (a naive treatment effect of 32.4%). For an increase in slope for both the control group (*γ*_3_=1.05) and the treatment group (*γ*_4_=−0.05), the bias increases by 161.5% (treatment participants have 48% less sodium at follow-up as compared to control participants).

**Fig. 3:**
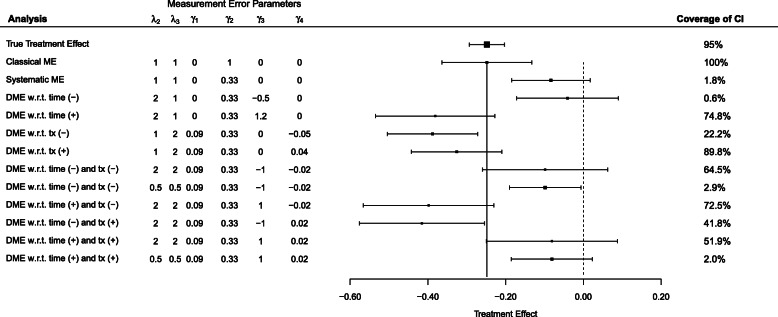
Forest plot of treatment effects, their associated 95% confidence intervals, and coverage of those intervals for the true treatment effect (top row), and naive treatment effects for a range of analyses based on altering the measurement error parameters in Table 2. The (+) and (−) refer to whether the *γ*_3_ and *γ*_4_ parameters are greater than or less than 0, respectively. The vertical solid line shows the true effect. The vertical dotted line at 0 indicates no treatment effect. In all analyses, *λ*_1_=1.86 and the sample size is *n*=372 per group

Figure 3 displays a forest plot of estimates of the naive treatment effect, their associated 95% confidence intervals, and the coverage of the confidence interval of the treatment effect under a range of different measurement error parameters. The vertical solid line shows the true treatment effect of −0.25. The vertical dotted line at 0 indicates no treatment effect.

Under classical measurement error, the estimate of the treatment effect is unbiased, but has increased variability. The (+) and (−) refer to whether the *γ*_3_ and *γ*_4_ parameters governing measurement error in Table 2 are greater than or less than 0, respectively. Bias in the treatment effect as well as increased variability occurs in systematic measurement error and differential measurement error with respect to time, treatment, or both time and treatment. Under some scenarios (systematic ME, DME w.r.t. time (-), DME w.r.t. time (-) and tx (-), DME w.r.t. time (+) and tx (+)), the bias and increased variability can be so great that the 95% confidence interval contains 0, such that the naive treatment effect is no longer significant. Under other scenarios, the bias is in the opposite direction, so that the naive treatment effect is greater than the true effect.

**Fig. 4:**
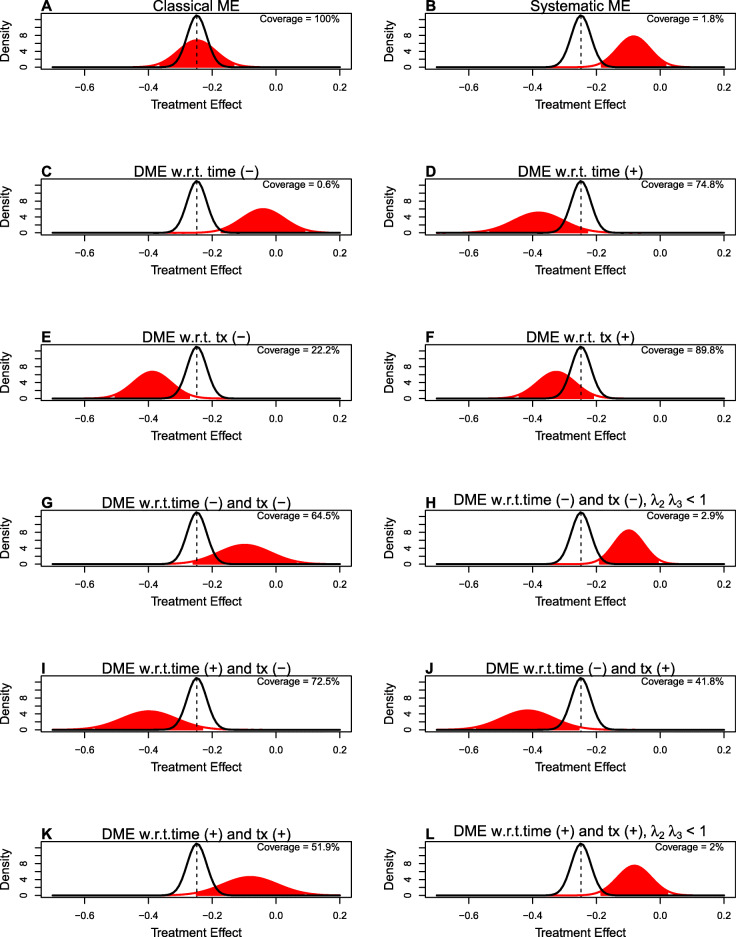
Density plots for the distribution of the naive treatment effect under different scenarios of measurement error (in red) compared to the true treatment effect (in black). Each panel A-J represents a different measurement error scenario. The dashed lines indicates the mean true treatment effect. The 95% confidence interval of the naive treatment effect is the shaded area under the curve (in red). Measurement error parameters for each scenario are equal to values found in Fig. 3. Coverage of the true treatment effect is 95%. In all analyses, the sample size is *n*=372 per group

Figure 4 displays density plots for the distribution of the treatment effect comparing the true treatment effect (in black) and the naive treatment effect under different scenarios of measurement error (in red). This provides a graphical illustration of coverage of the confidence interval of the treatment effect under the same measurement error-corrected scenarios in Fig. 3. Coverage of the true treatment effect is 95%. Under classical measurement error, the coverage is 100%. Coverage ranges from 0.6% to 89.8% depending on the differential measurement error scenario.

## Discussion

We found that when using self-reported dietary measures as outcomes in a lifestyle intervention study, differential measurement error with respect to treatment condition and time can result in a biased treatment effect and can impact the sample size needed to achieve 80% power in detecting a treatment effect. Increased variability in the outcomes measured with error (*y*_*ij*_), increases the sample size needed to achieve 80% power. The impact on sample size differs depending on the type of differential error: increased variability at follow-up (*λ*_2_) increases the required sample size at a faster rate than increased variability for treatment condition alone at follow-up (*λ*_3_). This is because increasing *λ*_2_ affects all observations, while increasing *λ*_3_ affects only those in the treatment group. Similarly, decreasing *λ*_2_ and/or *λ*_3_, decreases the sample size required to reach 80% power, with *λ*_2_ decreasing the required sample size at a faster rate than *λ*_3_. Naturally, when both factors increased/decreased variability, that had the largest percent increase/decrease on the sample size needed to achieve 80% power. By ignoring the possibility of increased variability at follow-up, trials may be under-powered.

Bias of the treatment effect is also affected by differential measurement error but here, differential measurement error with respect to treatment has a greater impact than does differential measurement error with respect to time. There is little additional bias when there is additional change in slope for the control group (*γ*_3_). When we set the other parameters in the measurement error model to 0 or 1 (*γ*_1_,*γ*_4_=0 and *γ*_2_=1), then bias is equal to (1+*γ*_3_)*β*_2_. Thus small values of *γ*_3_ have little effect on bias. However, even a small increase in slope for the treatment group (*γ*_4_) can have a substantial impact on bias (see Eq. 5).

In our simulations for power and bias, we fixed *γ*_1_, the additive difference in measurement error between treatment conditions at follow-up. Since *γ*_1_ does not appear in the variance calculations (see Eqs. 26 and 27) and only appears in the estimation of the naive treatment effect (*Ψ*^*naive*^), it will affect the sample size itself, but not the percent increase. For smaller values of *γ*_1_, the required sample size needed is smaller and for larger values of *γ*_1_, the required sample size is larger. When varying *γ*_1_ and keeping the other *γ* parameters constant at the TOHP values, the percent increase in sample size is still approximately 32.5*%*, 65%, and 130% when (*λ*_2_=1,*λ*_3_=2), (*λ*_2_=2,*λ*_3_=1), and (*λ*_2_=2,*λ*_3_=2) respectively. In terms of bias, *γ*_1_ is a constant term, shifting the naive treatment effect by the same amount across all values of *γ*_2_ and *γ*_3_ as it increases or decreases. Thus the shape of the contour plot in Fig. 2 stays the same, only its values change.

Similarly, *ρ*, the correlation between baseline and follow-up, was fixed throughout the simulations. The correlation *ρ* does not affect bias. As *ρ* increases, the sample size required to achieve 80% power decreases and as *ρ* decreases, the sample size must increase.

There is a relationship between the values of *λ*_2_,*λ*_3_ and the percent increase in sample size required to achieve 80% power. Figure 1 reports the percent increase in sample size relative to a referent scenario (*λ*_2_=1,*λ*_3_=1). Given the calculation of the sample size in Eq. 36, the ratio of sample sizes is equal to the ratio of the variances (Eq. 37). Solving Eq. 37 for a set of *λ* parameters, one can directly calculate the percent increase in sample size needed under scenarios of increased variability. As Eq. (39) makes clear, values of *λ*_2_ have both additive and multiplicative effects on the increase in sample size. This is why there is a doubling in the percent increase in sample size in Fig. 1 as the *λ* parameters change from (*λ*_2_=1,*λ*_3_=2, 32.5% increase), (*λ*_2_=2,*λ*_3_=1, 65% increase), (*λ*_2_=2,*λ*_3_=2, 130% increase).

The required sample size to achieve 80% power also depends on the *γ* parameters (Eq. 36) However, the ratio of the percent increase in sample size relative to the referent scenario for two different differential measurement error scenarios is invariant to the values of the *γ* parameters (Eq. 39).

Depending on the size and the sign of the measurement error parameters, the bias can be in the positive direction, towards zero, or in the negative direction. The combination of positive bias and increased variability can make the estimate of the treatment effect overlap with zero, resulting in a non-significant observed treatment effect. This can be seen in Fig. 3 under Systematic ME, as well as several differential measurement error scenarios where the 95% confidence interval crosses the dotted vertical line at zero. Bias in the negative direction (seen in estimates of the treatment effect to the left of the solid vertical line in Fig. 3) can make the treatment effect appear much larger than the true effect, which could lead investigators to think the intervention was much more successful than it actually was in reality. An extreme case of bias (not shown in Fig. 3) would not only bias in the positive direction, but show a significant positive effect, greater than zero. The treatment effect would be significant but in the opposite direction, and thus yielding a wrong conclusion.

Coverage of the treatment is also affected by differential measurement error. Coverage tends to be higher when there is less bias in the treatment effect measured with error, but increased variability, such as DME w.r.t. tx (+) in Fig. 3 (Panel F in Fig. 4). Although the true treatment effect is contained within the confidence interval of the naive effect when coverage is high, the naive estimate is highly variable. Coverage is low when there is a large bias, even if variability is increased. When coverage is low, the naive treatment effect differs greatly from the true treatment effect, due to bias. Coverage decreases when *λ*_2_ and *λ*_3_<1, due to decreased variability. This can be seen comparing panels G and H, and comparing panels K and L in Fig. 4.

In lifestyle intervention studies, it has been shown that measurement error in self-reported dietary measures can differ both with respect to treatment condition and over time. Natarajan et al. [7] investigated measurement error of dietary self-report in an intervention trial in which self-reported and plasma carotenoid biomarker data were available on all participants at each time point. Using a model which took into account measurement error in both self-report and biomarker-based measures, they found that self-reported accuracy improved in participants randomized to the intervention condition. They also found increases in variability among follow-up measurements in the intervention condition.

Espeland et al. [4] fit a longitudinal model to self-reported and urinary sodium to longitudinal data from a lifestyle intervention trial of 900 individuals with hypertension who were randomized to one of four conditions. They found that self-reported sodium intake was less than urinary sodium at all visits and within each study group. Interestingly, the ratio of self-reported to urinary sodium intake was smallest at follow-up compared to baseline in the most intensive intervention condition. The authors hypothesized that this was due to compliance bias and noted that, “subjective pressures to please staff and meet intervention goals led to under-reporting intakes.” Also, unlike the analyses by Natarajan et al. [7], measurement errors were *less* variable during follow-up than at baseline for all cohorts. This was attributed to better recall of foods containing sodium based on knowledge gained from the interventions.

Together, these results suggest that the presence of differential measurement error is likely to be intervention specific and may depend on the population being studied. For example, in a intervention study of youth with type 1 diabetes, Sanjeevi et al. [12] found no evidence of differential measurement error.

While our findings demonstrate the impact of differential measurement error, there are some limitations to this work. We only used two time points in developing our models. As the number of time points increases, so does the number of differential measurement error scenarios. We assumed continuous, normally-distributed outcomes. We used a linear measurement model as this is a common approach for modeling measurement error and empirically has been shown to provide a good fit to the data when both true intake and its version measured with error are available [6, 7, 13], especially after values have been log-transformed. In practice, investigators are often interested outcomes such as number of fruits and vegetables [14], which are not normally distributed and the impact of non-differential measurement error could be different. Future work will look at the impact of differential measurement error in non-normal outcomes. We based our simulations on self-reported sodium intake using parameters from the TOHP study. Examining measurement error for other components of diet, such as total intake, would require different parameter values, although one would expect to see results similar to those presented here. Finally, we focused on the setting of measurement error in dietary interventions. However, differential measurement error with respect to treatment and/or time can also exist in observational studies and an area of future work is to better understand the role of measurement error when estimating treatment effects using observational data.

## Conclusions

When designing a longitudinal lifestyle intervention study, researchers using self-reported dietary measures need to consider the impact of measurement error and differential measurement error. Recruiting a larger sample size can help overcome the loss of power associated with the additional variability due to measurement error. However, this approach does nothing to correct for bias. A more expansive approach that would allow the researcher to diagnose and correct for both bias and variance due to measurement error is to include an internal validation study with recovery biomarkers and implement methods that allow for measurement correction using internal validation studies [9, 15]. When an internal validation study is not possible, methods that use external validation studies [16, 17] are possible although they require the user to make additional assumptions regarding transportability of the measurement error model [18]. Still, we feel that these additional efforts to correct for measurement error are worthwhile, as they require only marginally more effort than conducting the intervention itself, and allow researchers to make inferences with greater accuracy and precision.

## Appendix

We present formulas for the bias of the naive treatment effect, its variance, and the coverage of its 95% confidence interval.

### Bias

Let *Z*_0_ refer to true intake at baseline, and *Z*_1_ refer to true intake at follow-up. Based on Eq. (1) in the main text, the mean value of true intake for each treatment condition and time point is given by

6 $$\begin{array}{*{20}l} \mathrm{E}(Z_{0} \mid d_{i}) &= \beta_{0}  \end{array} $$

7 $$\begin{array}{*{20}l} \mathrm{E}(Z_{1} \mid d_{i}=0) &= \beta_{0} + \beta_{1}  \end{array} $$

8 $$\begin{array}{*{20}l} \mathrm{E}(Z_{1} \mid d_{i}=1) &= \beta_{0} + \beta_{1} + \beta_{2}. \end{array} $$

Using Eqs. (6) through (8) the expected change in true intake in the control condition is

9 $$ \mathrm{E}\left[Z_{1} - Z_{0} \mid d_{i} = 0\right] = \beta_{1}  $$

and the expected change in true intake in the treatment condition is

10 $$ \mathrm{E}\left[Z_{1} - Z_{0} \mid d_{i} = 1\right] = \beta_{1} + \beta_{2}  $$

so that the expected true treatment effect is

11 $$ \mathrm{E}\left[Z_{1} - Z_{0} \mid d_{i}=1\right] - \mathrm{E}\left[Z_{1} - Z_{0} \mid d_{i}=0\right] = \beta_{2}.  $$

Let *Y*_*t*_ refer to self-reported intake at time *t*, *t*=0,1. The mean value of self-reported intake for each treatment condition and time point is given by

$$\mathrm{E}\left(Y_{t} \mid d\right) = \mathrm{E}_{z}\left[\mathrm{E}_{Y|Z}(y_{t} \mid z_{t}, d)\right] $$

so that, using Eq. (3) in the main text, the mean self-reported values by time and treatment group are:

12 $$\begin{array}{*{20}l} {}\mathrm{E}(Y_{0} \mid d_{i}=0) &= \gamma_{0} + \gamma_{2}\beta_{0}  \end{array} $$

13 $$\begin{array}{*{20}l} {}\mathrm{E}(Y_{0} \mid d_{i}=1) &= \gamma_{0} + \gamma_{2}\beta_{0}  \end{array} $$

14 $$\begin{array}{*{20}l} {}\mathrm{E}(Y_{1} \mid d_{i}=0) &= \gamma_{0} + \left(\gamma_{2} + \gamma_{3}\right) \left(\beta_{0} + \beta_{1}\right)  \end{array} $$

15 $$\begin{array}{*{20}l} {}\mathrm{E}(Y_{1} \mid d_{i}=1) &= \gamma_{0} + \gamma_{1} + \left(\gamma_{2} + \gamma_{3} + \gamma_{4}\right) \left(\beta_{0} + \beta_{1} + \beta_{2}\right). \end{array} $$

Using Eqs. (12) through (15), the expected change in self-reported intake in the control condition is

16 $$ \mathrm{E}[Y_{1} - Y_{0} \mid d_{i} = 0] = (\gamma_{2} + \gamma_{3})\beta_{1} + \gamma_{3} \beta_{0}  $$

sand the expected change in self-reported intake in the treatment condition is

17 $$\begin{array}{*{20}l} {}\mathrm{E}[Y_{1} - Y_{0} \mid d_{i} = 1] &= \gamma_{1} + \left(\gamma_{2} + \gamma_{3} + \gamma_{4}\right)\left(\beta_{0} + \beta_{1} + \beta_{2}\right)\\&\quad - \gamma_{2} \beta_{0}. \end{array} $$

The expected treatment effect from the self-reported intake (i.e. the naive treatment effect) is given by

18 $$\begin{array}{*{20}l} \Psi^{naive} &=\mathrm{E}[Y_{1} - Y_{0} \mid d_{i}=1] - \mathrm{E}[Y_{1} - Y_{0} \mid d_{i}=0] \notag \\ &= \gamma_{1} + \beta_{2}(\gamma_{2} + \gamma_{3}) + \gamma_{4}(\beta_{0} + \beta_{1} + \beta_{2}). \end{array} $$

The mean bias of the naive treatment effect is obtained by subtracting Eq. (11) from Eq. (18) to obtain.

19 $$ {}\text{Bias}(\Psi^{naive})=\gamma_{1} + \beta_{2}\left(\gamma_{2} + \gamma_{3}\right) + \gamma_{4}\left(\beta_{0} + \beta_{1} + \beta_{2}\right) - \beta_{2}.  $$

### Variance

The variance of self-reported intake for each treatment condition and time point is given by

$$ \text{Var}[Y_{t} \mid d] = \mathrm{E}[\text{Var}(Y_{t} \mid Z_{t}, d)] + \text{Var}[\mathrm{E}(Y_{t} \mid Z_{t}, d)] $$

so that,

20 $$\begin{array}{*{20}l} \text{Var}[Y_{0} \mid d_{i}=0] &= \sigma^{2}_{z}(\lambda_{1} + \gamma_{2}^{2}) \end{array} $$

21 $$\begin{array}{*{20}l} \text{Var}[Y_{0} \mid d_{i}=1] &= \sigma^{2}_{z}(\lambda_{1} + \gamma_{2}^{2}) \end{array} $$

22 $$\begin{array}{*{20}l} \text{Var}[Y_{1} \mid d_{i}=0] &= \sigma^{2}_{z}\left(\lambda_{1} \lambda_{2} + (\gamma_{2} + \gamma_{3})^{2}\right) \end{array} $$

23 $$\begin{array}{*{20}l} \text{Var}[Y_{1} \mid d_{i}=1] &= \sigma^{2}_{z}\left(\lambda_{1} \lambda_{2} \lambda_{3} + (\gamma_{2} + \gamma_{3} + \gamma_{4})^{2}\right). \end{array} $$

The covariance between baseline and follow-up self-reported intake is

$$\begin{array}{*{20}l} \text{Cov}(Y_{1}, Y_{0}) &= \mathrm{E}\left[\text{Cov}(Y_{1}, Y_{0} \mid Z_{1}, Z_{0})\right] \\&\quad+ \text{Cov}\left[\mathrm{E}(Y_{1} \mid Z_{1}, Z_{0}), \mathrm{E}(Y_{0} \mid Z_{1}, Z_{0})\right] \end{array} $$

so that, for the control condition (*d*_*i*_=0), the covariance is

24 $$\begin{array}{*{20}l} \text{Cov}(Y_{1}, Y_{0} \mid d_{i}=0) &= \rho \sigma^{2}_{z} + \text{Cov}[(\gamma_{0} + \gamma_{2}z_{{ij}}),\\&\quad (\gamma_{0} + (\gamma_{2} + \gamma_{3})z_{{ij}})] \notag \\ &= \rho \sigma^{2}_{z} + \gamma_{2}(\gamma_{2} + \gamma_{3}) \text{Cov}(z_{{ij}}, z_{{ij}}) \notag \\ &= \rho\sigma^{2}_{z}\left(1 + \gamma_{2}(\gamma_{2} + \gamma_{3})\right), \end{array} $$

and for the intervention condition (*d*_*i*_=1), the covariance is

25 $$\begin{array}{*{20}l} {}\text{Cov}(Y_{1}, Y_{0} \mid d_{i}=1) &= \rho \sigma^{2}_{z} + \text{Cov}\left[(\gamma_{0} + \gamma_{2}z_{{ij}}),\right.\\&\left. (\gamma_{0} + \gamma_{1} + (\gamma_{2} + \gamma_{3} \gamma_{4})z_{{ij}})\right]  \\ &= \rho \sigma^{2}_{z} \!+ \gamma_{2}(\gamma_{2} + \gamma_{3} + \gamma_{4}) \text{Cov}(z_{{ij}}, z_{{ij}}) \notag \\ &= \rho \sigma^{2}_{z}\left(1 + \gamma_{2}(\gamma_{2} + \gamma_{3} + \gamma_{4})\right). \end{array} $$

The variance in self-reported intake in the control condition is given by

26 $$\begin{array}{*{20}l} {}\text{Var}(Y_{1} - Y_{0} \mid d_{i} = 0) &= \text{Var}(Y_{1} \mid d_{i} = 0)\\& + \text{Var}(Y_{0} \mid d_{i} = 0) - 2\text{Cov}(Y_{0}, Y_{1}) \notag \\ &= \sigma^{2}_{z}\left(\lambda_{1}\lambda_{2} + (\gamma_{2} + \gamma_{3})^{2} + \lambda_{1} + \gamma_{2}^{2} \notag \right.\\ &-\left. 2\rho(1+ \gamma_{2}(\gamma_{2} + \gamma_{3}))\right). \end{array} $$

The variance in self-reported intake in the treatment condition given by

27 $$\begin{array}{*{20}l} \text{Var}(Y_{1} - Y_{0} \mid d_{i} = 1) &= \sigma^{2}_{z}\left(\lambda_{1}\lambda_{2}\lambda_{3} + (\gamma_{2} + \gamma_{3} + \gamma_{4})^{2} + \lambda_{1} + \gamma_{2}^{2}- 2\rho(1 + \gamma_{2}(\gamma_{2} + \gamma_{3} + \gamma_{4}))\right). \end{array} $$

### Coverage

The coverage probability of a confidence interval is the proportion of the time that the interval contains the true quantity of interest. From Eq. (11), the true treatment effect is equal to *β*_2_.

For either treatment condition (*d*_*i*_=0,1), let *z* ¯_1_ be the sample mean of *z* at time 1 and let *z* ¯_0_ be the sample mean of *z* at time 0. Let *ρ* be the correlation between *z*_0_ and *z*_1_ and let *n*_*d*_ be the sample size in either treatment condition. Regardless of treatment condition, the variance of *z* ¯_1_−*z* ¯_0_ is defined as

28 $$\begin{array}{*{20}l} \text{Var}(\Bar{z}_{1} - \Bar{z}_{0}) &= \frac{\sigma^{2}_{z}}{n_{d}} + \frac{\sigma^{2}_{z}}{n_{d}} - 2 \frac{\rho \sigma^{2}_{z}}{n_{d}}\notag \\ & = \frac{2\sigma^{2}_{z}(1-\rho)}{n_{d}}. \end{array} $$

Let *δ*_1_=*z* ¯_1_−*z* ¯_0_ for *d*_*i*_=1 and let *δ*_0_=*z* ¯_1_−*z* ¯_0_ for *d*_*i*_=0. The estimate of the treatment effect *β*_2_ is $\hat {\beta }_{2} = \delta _{1} - \delta _{0}$. The variance of the estimated treatment effect is

29 $$ \text{Var}(\hat{\beta}_{2}) = \frac{4\sigma^{2}_{z}(1-\rho)}{n_{d}}  $$

So that assuming Normality as in (1) in the main text, the sampling distribution of $\hat {\beta }_{2}$ is

30 $$ \hat{\beta}_{2} \sim N \left(\beta_{2}, \frac{4\sigma^{2}_{z}(1-\rho)}{n_{d}}\right).  $$

Let *y* ¯_1_ be the sample mean of *y* at time 1 and let *y* ¯_0_ be the sample mean of *y* at time 0. Let $\delta ^{\ast }_{1} = \Bar {y}_{1} - \Bar {y}_{0}$ for *d*_*i*_=1 and let $\delta ^{\ast }_{0} = \Bar {y}_{1} - \Bar {y}_{0}$ for *d*_*i*_=0. An estimate of the naive treatment effect is $\hat {\Psi }^{naive} = \delta ^{\ast }_{1} - \delta ^{\ast }_{0}$. Its variance is

31 $$ {}\text{Var}(\hat{\Psi}^{naive}) = \frac{\text{Var}(Y_{1} - Y_{0} \mid d_{i}=1)}{n_{d}} + \frac{\text{Var}(Y_{1} \!- Y_{0} \mid d_{i}=0)}{n_{d}}  $$

where the variance terms in the numerator were defined in Eqs. (26) and (27).

A 95% confidence interval for the naive treatment effect is:

32 $$ \hat{\Psi}^{naive} \pm 1.96 {\ast} \text{SE}(\hat{\Psi}^{naive})  $$

The coverage of this confidence interval is the probability that it contains the true quantity of interest

33 $$ \text{Coverage}= Pr(\Psi_{{lower}} < \hat{\beta}_{2} < \Psi_{{upper}})  $$

where *Ψ*_*lower*_ and *Ψ*_*upper*_ are the endpoints of the confidence interval in (32).

### Power and sample size

We power based on a two-sample z-test for the difference in mean change scores between treatment and control groups. The difference in means under the alternative hypothesis is the naive treatment effect *Ψ*^*naive*^, given by Eq. (18). The non-centrality parameter is

34 $$ \Delta = \frac{\Psi^{naive}} {\sqrt{\text{Var}(\hat{\Psi}^{naive})}}  $$

where the denominator is the square root of the variance in Eq. (31). Under a two-sample z-test, the critical value under the two-sided null hypothesis at a Type 1 error rate of.05 is -1.96, so that power is calculated by

35 $$ \text{Power} = \Phi(-1.96 - \Delta),  $$

where *Φ* represents the standard normal distribution function.

Solving Eq. (35 for *n*_*d*_, we can obtain an equation for the sample size of each treatment group. Assuming power of 80% and a Type 1 error rate of.05, the sample size of each group is given by

36 $$\begin{array}{*{20}l} n_{d} &= (\Phi^{-1}(.80) + 1.96)^{2} \\&\quad\times \frac{\text{Var}(Y_{1} - Y_{0} \mid d_{i} = 1) + \text{Var}(Y_{1} - Y_{0} \mid d_{i} = 0)}{(\Psi^{naive})^{2}} \end{array} $$

The total sample size required is thus 2*n*_*d*_.

Figure 1 reports the percent increase in sample size relative to a referent scenario. Let *n* be the sample size under the referent scenario and let *n*^∗^ be the sample size under an alternative scenario with values $\lambda _{2}^{\ast }, \lambda _{3}^{\ast }$. The proportion increase in sample size reported in Fig. 1 is $\frac {n^{\ast }}{n}-1$ or $\frac {n^{\ast } - n}{n}$. Using Eq. (36, the ratio of sample sizes is equal to the ratio of variances.

37 $$\begin{array}{*{20}l} {}\frac{n^{\ast}}{n} &= \frac{\text{Var}^{\ast}(Y_{1} - Y_{0} \mid d_{i} = 1) + \text{Var}^{\ast}(Y_{1} - Y_{0} \mid d_{i} = 0)}{\text{Var}\left(Y_{1} - Y_{0} \mid d_{i} = 1\right) + \text{Var}\left(Y_{1} - Y_{0} \mid d_{i} = 0\right)}\notag \\ &= \frac{\left(\lambda_{2}^{\ast}+2+\lambda_{2}^{\ast}\lambda_{3}^{\ast}\right)+c}{\left(\lambda_{2}+2+\lambda_{2}\lambda_{3}\right)+c} \end{array} $$

where *c* is a constant term that does not depend on any values of *λ*. The ratio of percent increases under two different scenarios, for example, non-differential measurement error with respect to treatment and non-differential measurement error with respect to time is:

38 $$ \left(\frac{n^{\ast} - n}{n^{\ast\ast} - n}\right) = \frac{\lambda_{2}^{\ast}(1 + \lambda_{3}^{\ast}) - \lambda_{2}(1 + \lambda_{3})} {\lambda_{2}^{\ast\ast}(1 + \lambda_{3}^{\ast\ast}) - \lambda_{2}\left(1 + \lambda_{3}\right)}.  $$

Figure 1 used non-differential measurement error with respect to treatment and time as the reference scenario (*λ*_2_=1,*λ*_3_=1). Under this reference scenario, Eq. (38 reduces to

39 $$ \left(\frac{n^{\ast} - n}{n^{\ast\ast} - n}\right) = \frac{\lambda_{2}^{\ast}\left(1 + \lambda_{3}^{\ast}\right) - 2} {\lambda_{2}^{\ast\ast}\left(1 + \lambda_{3}^{\ast\ast}\right) - 2}.  $$

## Data Availability

The simulation parameter values were calibrated using data on sodium intake from the Trials of Hypertension Prevention Study (TOHP). The data is available upon request at https://biolincc.nhlbi.nih.gov/studies/tohp/.

## Abbreviations

ME: measurement error
DME: differential measurement error
w.r.t.: with respect to
tx: treatment
CI: confidence interval
TOHP: Trials of Hypertension Prevention Study
